# Efficacy of left atrial low-voltage area-guided catheter ablation of atrial fibrillation: An updated systematic review and meta-analysis

**DOI:** 10.3389/fcvm.2022.993790

**Published:** 2022-11-17

**Authors:** Yaqiong Zhou, Huamin Zhang, Peng Yan, Peng Zhou, Peijian Wang, Xiaoping Li

**Affiliations:** ^1^Department of Cardiology, The First Affiliated Hospital of Chengdu Medical College, Chengdu, China; ^2^Key Laboratory of Aging and Vascular Homeostasis of Sichuan Higher Education Institutes, Chengdu, China; ^3^Sichuan Clinical Research Center for Geriatrics, The First Affiliated Hospital, Chengdu, China; ^4^Department of Epidemiology and Statistics, Chengdu Medical College, Chengdu, China; ^5^Department of Cardiology, Sichuan Provincial People’s Hospital, University of Electronic Science and Technology of China, Chengdu, China

**Keywords:** atrial fibrillation, catheter ablation, pulmonary vein isolation, low-voltage areas, recurrence

## Abstract

**Aims:**

This study aimed to evaluate the efficacy of low-voltage area (LVA)-guided substrate modification catheter ablation in patients with atrial fibrillation (AF).

**Methods:**

Systematic searches of the PubMed, EMBASE, and Cochrane databases were performed from inception to July 2022 for all available studies. The effect estimates were combined with the Mantel–Haenszel random-effects model. Subgroup analyses, sensitivity analysis, and meta-regression were conducted to explore the sources of statistical heterogeneity.

**Results:**

A total of 16 studies involving 1942 subjects (mean age: 61 ± 10 years, 69% male) were identified. All studies included patients with paroxysmal AF, non-paroxysmal AF, or both. At a mean follow-up of 18.9 months, patients who underwent LVA-guided substrate modification ablation had significantly higher freedom from all-atrial tachycardia recurrence than patients who underwent control ablation [67.7% vs. 48.9%, risk ratios (RR) 0.64, 95% confidence interval (CI) 0.55–0.76, *P* < 0.001], with 36% relative risk and 18.7% absolute risk reductions in all-atrial tachycardia recurrence. Subgroup analysis based on AF types demonstrated that the decreased risk of all-atrial tachycardia recurrence was present predominantly in non-paroxysmal AF compared with paroxysmal AF (RR 0.60, 95% CI 0.52–0.69 vs. RR 0.96, 95% CI 0.81–1.13).

**Conclusion:**

Low-voltage area-guided substrate modification ablation combined with PVI appears to have a significant beneficial effect of improving freedom from all-atrial tachycardia recurrence, especially in patients with non-paroxysmal AF.

## Introduction

Atrial fibrillation (AF), one of the most frequent chronic arrhythmias, has a prevalence of 0.4–1.0% in the general population and is associated with a threefold increased risk of heart failure, fivefold increased risk of stroke, and twofold increased risk of all-cause mortality. Catheter ablation using the technique of pulmonary vein isolation (PVI) has emerged as a first-line treatment strategy for patients with paroxysmal AF, but PVI alone is much less successful in non-paroxysmal AF, with a reported 2-year AF freedom rate of 40–50% (1–4). Although several additional ablation strategies, including substrate modification, have been developed, to date, there have been no uniform approaches revealing additional benefit over PVI alone (5, 6).

It is generally acknowledged that atrial structural remodeling involving atrial tissue fibrosis and scarring is an essential factor in the pathogenesis and progression of AF (7, 8). Several trials have suggested that the existence of LVA was strongly associated with AF recurrence after catheter ablation for all AF types (9–12). In this context, in Rolf et al. initially proposed LVA-guided ablation as an atrial substrate modification approach (9). To date, numerous studies have investigated the feasibility and effectiveness of LVA-guided substrate modification in AF ablation given the theoretical and practical rationality (13–15). However, their results were surprisingly conflicting. In addition, most of the identified studies were single-center studies with small sample-sizes, which compromised the credibility of the results (16). In 2017, a previously published meta-analysis from Blandino et al. suggested that LVA ablation in addition to PVI was superior to the traditional technique without a significant increase in rates of adverse events (17). However, only six studies with one randomized trial met the inclusion criteria, and high heterogeneity might affect the interpretation of results. Moreover, several additional articles were reported after this systematic review, especially some randomized trials revealing conflicting results. Against this background, we conducted a comprehensive meta-analysis of all the available studies to assess the efficacy of LVA-guided substrate modification catheter ablation of AF.

## Methods

### Search strategy

The study followed the guidelines specified in the Preferred Reporting Items for Systematic Review and Meta-Analysis Protocols (PRISMA-P) (18). Systematic searches of the PubMed, EMBASE, and Cochrane databases were conducted from database inception to July 2022. The search terms included “atrial fibrillation,” “catheter ablation,” “pulmonary vein isolation,” “low voltage areas,” and “recurrence.” We searched for articles without language restriction, and the reference lists of possible eligibility were also reviewed.

### Selection criteria

Studies were considered eligible if (1) they had a prospective or retrospective design. (2) The study population was composed of AF patients undergoing voltage mapping and catheter ablation. (3) Comparisons were conducted between the study group (LVA ablation plus PVI) and the control group (PVI strategy). (4) The outcome was AF or all-atrial tachycardia recurrence. (5) The follow-up duration to determine outcomes was at least 6 months. (6) Enough data were provided to assess risk ratios (RR). In the case of duplicate reporting on the same subjects, the largest sample or most comprehensive information was selected.

### Data extraction and quality assessment

Data were extracted by two independent investigators (YZ and HZ) using a standardized data extraction form. The form included the following characteristics of each study: first author’s name, year of publication, study design, sample size, geographical location, mean age, sex, AF type, left atrial diameter (LAD), left ventricular ejection fraction (LVEF), treatment group ablation schemes, control group ablation schemes, cutoff of LVA, rhythm during voltage mapping [sinus rhythm (SR) or AF], procedure time, radiofrequency ablation time, radiofrequency power settings, type of 3D mapping system, type of high density mapping catheter, number of points mapped, endpoint of LAV-guided ablation, antiarrhythmic drugs (AADs) after ablation, follow-up duration, and outcomes. Disagreements between the two independent investigators were resolved by consensus.

The methodological quality of eligible trials was evaluated by two authors (YZ and HZ) using the Cochrane Handbook for Systematic Reviews of Interventions Criteria (19) for randomized controlled trials (RCT) and the Newcastle–Ottawa Scale (NOS) (20). Quality Assessment Scale for cohort studies. Discrepancies were resolved by a third reviewer (PY).

### Outcome measures

The primary outcome was AF or all-atrial tachycardia recurrence during follow-up. Holter monitoring was used to detect arrhythmia, defined by an AF or all-atrial tachycardia episode of at least 30 s duration.

### Statistical analysis

The overall effect estimates of all outcomes analyzed by RR and their 95% confidence intervals (CI). The effect estimates were combined with the Mantel–Haenszel random-effects model because heterogeneity was anticipated among studies using a profile likelihood model in Stata/MP 14 (Stata Corp.). Between-group heterogeneity was assessed using the *I*^2^ statistic. We considered *I*^2^ values < 25% as low heterogeneity and >75% as high heterogeneity. The sources of heterogeneity were investigated by subgroup analyses, sensitivity analysis, and meta-regression.

We further evaluated whether the benefit of LVA-guided substrate modification ablation depended on features such as study design (RCT vs. non-RCT), AF type (paroxysmal AF, non-paroxysmal AF, or both types), rhythm during voltage mapping (during SR vs. AF), the sequence of ablation and mapping (ablation before mapping vs. ablation after mapping), cutoff of LVA (≤0.5 mV vs. 0.1–0.4 mV), follow-up duration (≤24 months vs. >24 months), LAD (≤45 mm vs. >45 mm), and ablation targets (LVA ablation vs. LVA + transitional zones ablation). A random-effects meta-regression model was applied to examine subgroup differences. Publication bias was assessed by visual symmetry of funnel plots and Egger’s and Begg’s tests. Statistical analyses were performed using STATA 14.0 (Stata Corp., College Station, TX, USA) and Review Manager version 5.3. A 2-tailed *P-*value ≤ 0.05 was considered statistically significant for all analyses except for subgroup interactions, for which the significance level was defined as *P* ≤ 0.10.

## Results

### Study selection

The systematic search identified 1,596 records that were screened by their titles and abstracts for possible eligibility after the duplicates were removed. After screening, 52 studies were identified as potentially relevant, and their full-text manuscripts were carefully reviewed for possible inclusion. Finally, 16 articles fulfilled the inclusion criteria and were analyzed in our meta-analysis (9, 13–15, 21–32) (Supplementary Figure 1).

### Study characteristics

Overall, 1942 patients (mean age of 61 ± 10 years, 69% male) from 16 studies [six RCT (13, 15, 21, 28–30), three prospective studies (9, 23, 26), and seven retrospective studies (14, 22, 24, 25, 27, 31, 32)] were identified. The sample sizes ranged from 50 to 229, and the mean duration of follow-up was 18.9 months, ranging from 9.3 to 48 months. Two studies (15, 26) enrolled 173 participants with paroxysmal AF, 11 studies recruited a total of 1,377 patients with non-paroxysmal AF, and the remaining three studies (9, 27, 29) included 392 participants with both AF types. In the study group, LVA ablation was performed after PVI in all trials, and the transitional zones were targeted for elimination in three studies (21, 25, 28). However, in the control group, ten studies involved traditional PVI, and six studies used PVI plus other ablation strategies, including stepwise ablation in three studies (21, 25, 28), box isolation in one study (30), and empirical linear ablation in two studies (29, 32). In the meta-analysis, two definitions of LVAs were adopted. Fourteen studies used a cutoff of LVA ≤ 0.5 mV as the qualification for a positive “LVA,” while the remaining two articles (25, 28) used a cutoff of LVA 0.1–0.4 mV. High-density mapping was performed during stable sinus rhythm in 12 of the 16 studies and during AF in the remaining three studies (13, 14, 27). The mean LAD and LVEF were 44 ± 7 mm and 57 ± 11%, respectively. All identified studies had a primary endpoint of all-atrial arrhythmia recurrence, and of these, one study (32) used AF or atrial flutter recurrence as the primary endpoint. Detailed information on the baseline characteristics of the included studies is summarized in Table 1.

**TABLE 1:** Study characteristics.

Author	Location	Design	Sample	Male (%)	Age (years)	AF type	Follow-up (moths)	LVA cutoffs	Rhythm during voltage mapping	LAD (mm)	LVEF (%)	Treatment group ablation schemes	Control group ablation schemes	Primary endpoints
Wang et al. (21)	China	RCT	124	76 (61)	63 ± 8	Non-paroxysmal AF	12	≤0.5 mV	During SR	45 ± 4	64 ± 5	PVI + LVA	PVI + stepwise	All-atrial tachycardia recurrence
Rolf et al. (9)	Germany	Prospective	73	40 (55)	67 ± 8	Paroxysmal AF and non-paroxysmal AF	12	≤0.5 mV	During SR	44 ± 7	59 ± 0	PVI + LVA	PVI	All-atrial tachycardia recurrence
Yang et al. (25)	China	Retrospective	164	126 (77)	54 ± 10	Non-paroxysmal AF	30	0.1–0.4 mV	During SR	42 ± 5	62 ± 7	PVI + LVA	PVI + stepwise	All-atrial tachycardia recurrence
Jadidi et al. (23)	Germany	Prospective	151	104 (70)	61 ± 9	Non-paroxysmal AF	13	≤0.5 mV	During AF or SR	45 ± 5	54 ± 9	PVI + LVA	PVI	All-atrial tachycardia recurrence
Cutler et al. (22)	USA	Retrospective	141	93 (66)	61 ± 11	Non-paroxysmal AF	12	≤0.5 mV	During SR	44 ± 6	49 ± 14	PVI + LVA	PVI	All-atrial tachycardia recurrence
Yamaguchi et al. (24)	Japan	Retrospective	55	35 (63)	64 ± 8	Non-paroxysmal AF	18	≤0.5 mV	During SR	44 ± 5	63 ± 12	PVI + LVA	PVI	All-atrial tachycardia recurrence
Yang et al. (28)	China	RCT	229	176 (77)	57 ± 9	Non-paroxysmal AF	18	0.1–0.4 mV	During SR	41 ± 5	62 ± 7	PVI + LVA	PVI + stepwise	All-atrial tachycardia recurrence
Yagishita et al. (27)	USA	Retrospective	201	113 (56)	65 ± 9	Paroxysmal AF and non-paroxysmal AF	12	≤0.5 mV	During AF	46 ± 8	50 ± 13	PVI + LVA	PVI	All-atrial tachycardia recurrence
Mohanty et al. (26)	USA	Prospective	111	79 (71)	60 ± 10	Paroxysmal AF	27	≤0.5 mV	During SR	40 ± 5	54 ± 7	PVI + LVA	PVI	All-atrial tachycardia recurrence
Kircher et al. (29)	Germany	RCT	118	77 (65)	63 ± 10	Paroxysmal AF and non-paroxysmal AF	12	≤0.5 mV	During SR	43 ± 6	60 ± 8	PVI + LVA	PVI + linear	All-atrial tachycardia recurrence
Kumagai et al. (30)	Japan	RCT	54	39 (72)	65 ± 9	Non-paroxysmal AF	24	≤0.5 mV	During SR	46 ± 5	61 ± 7	PVI + BOX + LVA	PVI + BOX	All-atrial tachycardia recurrence
Nery et al. (31)	Canada	Retrospective	145	117 (80)	61 ± 10	Non-paroxysmal AF	18	≤0.5 mV	During SR	42 ± 11	52 ± 6	PVI + LVA	PVI	All-atrial tachycardia recurrence
Masuda et al. (15)	Japan	RCT	62	18 (29)	75 ± 8	Paroxysmal AF	25	≤0.5 mV	During SR	39 ± 6	-	PVI + LVA	PVI	All-atrial tachycardia recurrence
Liu et al. (14)	China	Retrospective	136	105 (72)	58 ± 13	Non-paroxysmal AF	48	≤0.5 mV	During AF	-	-	PVI + LVA	PVI	All-atrial tachycardia recurrence
Hwang et al. (13)	Korea	RCT	50	43 (86)	58 ± 10	Non-paroxysmal AF	12	≤0.5 mV	During AF	49 ± 5	59 ± 11	PVI + LVA	PVI	All-atrial tachycardia recurrence
Suzuki et al. (32)	Japan	Retrospective	128	97 (76)	68 ± 11	Non-paroxysmal AF	9.3	≤0.5 mV	During SR	46 ± 6	59 ± 14	PVI + LVA	PVI + linear	AF or atrial flutter recurrence

LVA, atrial low-voltage areas; RCT, randomized controlled trials; AF, atrial fibrillation; SR, sinus rhythm; LAD, left atrial diameter; LVEF, left ventricular ejection fraction; PVI, pulmonary veins isolation.

In most of the included studies, the concept of LVA ablation was more dependent on LVA size. Smaller LVAs were approached by regional ablation aiming at tissue homogenization. Larger LVAs were targeted by linear ablation traversing the LVA and connecting non-excitable areas. Broader LVAs were approached by encircling the LVA at the border of normal voltage tissue. No between-group difference could be found with respect to the proportions of patients with LVA. The endpoint of LVA ablation was defined as the absence of local electrical potential using high output pacing at the ablation site or elimination of the local potentials. Total procedural time was reported in 14 studies (9, 13, 15, 21, 23–32), and there were no statistically significant differences between the LAV ablation group and the control group (194.24 min vs. 189.26 min, *P* = 0.15). Radiofrequency ablation time was reported in 12 studies (9, 13, 14, 21–23, 25–27, 29–31), and similarly, no between-group difference was found (62.69 ± 29.66 vs. 61.33 ± 31.59, *P* = 0.40). Power delivery was limited to 20–25 Watts on the posterior wall near the esophagus and to 25–48 W for the remaining left atrium regions. More than 500 points in the left atrium were acquired to create a voltage map in most of the included studies (15, 23–25, 28, 30–32). The CARTO3 system (13, 14, 21, 22, 26, 27, 31) and EnSite-NavX mapping system (24, 25, 28, 30, 32) were the most common 3D mapping systems. The majority of articles used HD grids (23–25, 28, 30, 32), pentaray catheters (15, 31), or Lasso-Nav catheters (13, 21, 26, 27, 29) as the mapping catheter. All patients without AADs after ablation were reported in five studies (13–15, 23, 32), and AADs were discontinued 3 months after ablation in seven studies (21, 22, 25, 26, 28–30), 1.5 months in one study (31), 3–6 months in one study (27), and 6 months in one study (24). Detailed information on the procedural characteristics of the included studies is shown in Supplementary Table 1.

### Quality assessment

**FIGURE 1:**
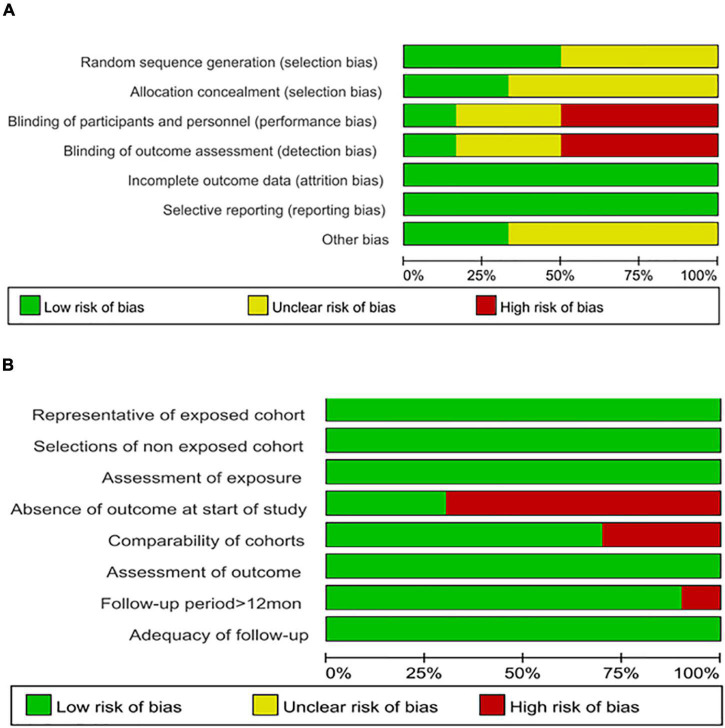
**(A)** Assessment of methodological quality of the included randomized studies using the Cochrane Risk of Bias Assessment Tool. **(B)** Assessment of methodological quality of the included cohort studies using the Newcastle–Ottawa Quality Assessment Scale. Stacked bars represent the proportion of studies with a high (red), unclear (yellow), or low (green) risk of bias and applicability concerns.

In general, all ten identified non-randomized articles were of high quality in terms of risk of bias based on the NOS assessment, with only one study (24) showing the lowest score of seven. However, the overall risk of bias of the six included randomized trials was deemed high for three trials (15, 21, 29) in terms of the performance bias and detection bias and unclear for three trials (13, 25, 30) because the information on random sequence generation, allocation concealment, and blinding of participants and personnel was unclear using the Cochrane Handbook for Systematic Reviews of Interventions criteria. All assessment results are shown in Figure 1.

### Overall analysis

**FIGURE 2:**
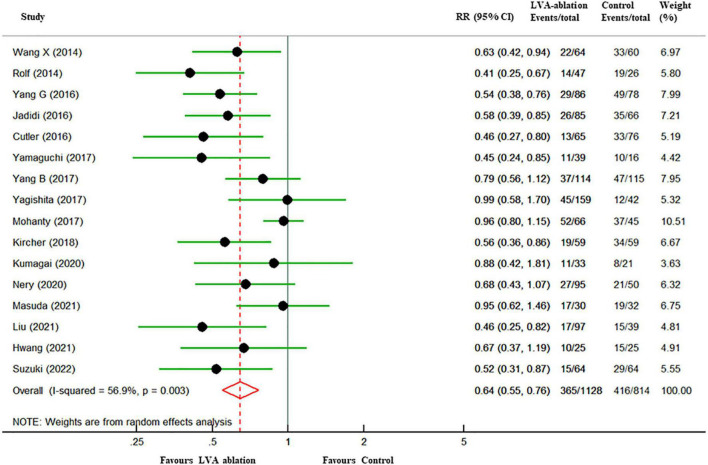
Forest plots of all-atrial tachycardia recurrence among patients with LVA-guided substrate modification ablation compared with those who underwent control ablation. The size of the circle-plotting symbol is proportional to the size of the study. Horizontal lines are the 95% CI. LVA, atrial low-voltage areas; RR, risk ratios; CI, confidence intervals.

In the overall analysis, at a mean follow-up of 18.9 months, freedom from all-atrial tachycardia recurrence in patients who underwent LVA ablation was significantly higher than that in those who underwent control ablation (67.7% vs. 48.9%, RR 0.64, 95% CI 0.55–0.76, *P* < 0.001), with moderate heterogeneity (*I*^2^ = 57%). A 36% relative risk reduction and 18.7% absolute risk reduction in all-atrial arrhythmia recurrence were yielded in the LVA ablation group (Figure 2). Six studies (13, 26, 28, 29, 31, 32) assessed the incidence of ischemic stroke or transient ischemic attack (TIA) between the two groups. However, no stroke or TIA occurred in most of these studies, and thus, we did not perform a pooled analysis.

### Subgroup analyses

Subgroup analysis based on study design revealed a slight decrease recurrence in non-RCT group (RR 0.59, 95% CI 0.46–0.76 vs. RR 0.73, 95% CI 0.61–0.87) with moderate heterogeneity in the former group (*I*^2^ = 72%) and significantly decreased heterogeneity in the latter group (*I*^2^ = 0%). In the six RCT studies, a further analysis stratified by control group ablation schemes (PVI only or PVI + adjunctive ablations) demonstrated an increased all-atrial tachycardia recurrence in PVI only group (RR 0.84, 95% CI 0.60–1.18 vs. RR 0.69, 95% CI 0.55–0.85) with a negligible level of heterogeneity in both group (Supplementary Figure 2).

Subgroup analysis based on AF types, the decreased risk of all-atrial tachycardia recurrence was present predominantly in non-paroxysmal AF group (RR 0.60, 95% CI 0.52–0.69) with *I*^2^ values of 0% and mixed AF group (RR 0.60, 95% CI 0.37–0.97) with *I*^2^ values of 66%. However, the RR did not reach statistical significance in the paroxysmal AF group (RR 0.96, 95% CI 0.81–1.13) with *I*^2^ values of 0%.

**FIGURE 3:**
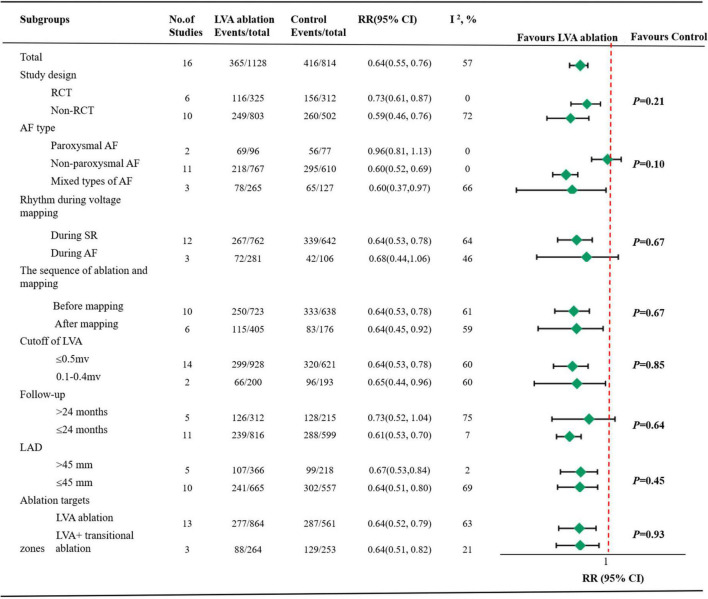
Pooled summary results by subgroups. LVA, atrial low-voltage areas; RR, risk ratios; CI, confidence intervals; RCT, randomized controlled trials; AF, atrial fibrillation; SR, sinus rhythm; LAD, left atrial diameter.

Subgroup analysis based on rhythm during voltage mapping, the sequence of ablation and mapping, cutoff of LVA, follow-up duration, LAD and ablation targets demonstrated comparable rhythm outcomes between the two groups. There were no differences noted among the subgroups except for the AF types based on the meta-regression analysis (Supplementary Table 2). All details are illustrated in Figure 3.

### Sensitivity analysis and publication bias

Sensitivity analysis showed that the overall results did not change significantly after excluding one study at a time, which suggested that the results of this study were statistically reliable (Supplementary Figure 3). Neither Begg’s funnel plot nor Egger’s test (*P* = 0.21) showed evidence of publication bias (Supplementary Figure 4).

## Discussion

In view of the increasing recognition of the arrhythmogenic role of LVA, we systematically assessed the evidence known on LVA-guided substrate modification ablation in addition to PVI in AF patients undergoing catheter ablation. The present meta-analysis, involving 1942 individuals from 16 studies, with a mean follow-up of 18.9 months, analyzed the long-term role of LVA-guided ablation. Several important findings were demonstrated as follows: (1) At a mean follow-up of 18.9 months, a 36% relative risk reduction and an 18.7% absolute risk reduction in all-atrial tachycardia recurrence were noted with LVA-guided substrate modification ablation compared to the traditional PVI strategy. (2) The effectiveness of LVA ablation was evident predominantly in non-paroxysmal AF patients.

In an attempt to improve ablation outcomes, more adjunctive substrate modification ablation approaches were frequently used, including continuous fractionated electrograms, left atrial appendage electrical isolation, or application of a roof or mitral isthmus line beyond PVI (33, 34), but the results did not provide additional benefits according to the available data (5, 35). The discouraging conclusion of these studies might suggest that the above ablation strategies possibly fail to address the specific mechanisms that were responsible for perpetuating AF in some patients. It is well recognized that atrial remodeling, including atrial fibrosis and scarring, plays a critical role in AF pathogenesis because conduction slows the predisposition to reentry (36). Several studies have shown that cardiac magnetic resonance imaging (MRI) is a promising technique to evaluate atrial fibrosis (37), but it is challenging in thin-walled atria (6). High-density voltage mapping of LVA serves as a reliable surrogate technique for the evaluation of atrial substrate since it correlates well with cardiac MRI scans. Several studies have explored the association between the severity of LVA and AF/AT recurrence. Most of them used a criterion of the LVA described as ≤0.5 or 0.4 mV covering > 10% or 5 cm^2^ of the total left atrial surface and demonstrated a causal relationship between increased LVA% and AF/AT recurrence (38, 39). These findings from the rationale for voltage-guided substrate modification suggest that ablation strategies targeting LVA might improve ablation outcomes. In the meta-analysis, 32.4% of patients in the LVA ablation group experienced all-atrial arrhythmia recurrence compared to 51.1% of those treated with PVI. The results indicated that this strategy was a low-potential proarrhythmic technique. Therefore, the LVA substrate ablation strategy provided the possibility of a personally tailored ablation approach based on the extent of atrial disease and could also be seen as guidance for identifying appropriate candidates for catheter ablation of AF, accounting for its excellent efficacy.

Usually, cerebral thromboembolism following AF ablation is an essential safety concern. An extensive linear ablation approach to isolating the LVA could compromise LA contractile function and lead to a prothrombotic state. In addition, it was noted that appropriate anticoagulant regimens were not used for every patient with indications. Among the studies included in the present meta-analysis, six studies reported the endpoints of cerebral thromboembolism, and no stroke/TIA occurred during the follow-up period in 5/6 studies either in the LVA ablation group or in the control group. In the study by Kircher et al. (29), stroke/TIA was observed in 0/59 patients in the LVA ablation group and 1/59 patients in the control group. Based on these data, it seemed that LVA ablation in addition to PVI did not sacrifice cerebral safety. However, due to the lack of stroke/TIA events, we did not perform pooled analysis. Further adequately powered RCT are needed to clarify the cerebral complications of LVA-guided catheter ablation of AF.

The benefits of LVA ablation were limited to patients with non-paroxysmal AF in the present meta-analysis. Two hypotheses might explain the different responses between paroxysmal AF and non-paroxysmal AF. First, the mechanisms of AF recurrence after ablation were substantially different in different AF types. Several studies have suggested that reconnection of the PV represents the dominant mechanism of all-atrial tachycardia recurrence in paroxysmal AF, whereas in non-paroxysmal AF, local re-entry and fibrillatory conduction related to atrial remodeling might be the dominant factor (40). Therefore, the efficacy of LVA ablation was greater in non-paroxysmal AF since ablation targeting LVA changed diseased conduction areas into scar areas with no electrical conduction. Second, the generation of LVAs in paroxysmal AF was more dependent on upstream factors causing atrial remodeling persistently even after ablation, such as atrial pressure, aging, and female sex (41, 42). However, non-paroxysmal AF was more likely to depend on the atrial substrate caused by AF burden than paroxysmal AF (43, 44). Accordingly, LVA ablation might not provide a good clinical effect if the LVA was not caused by the atrial substrate. It is worth noting that an ablation strategy cannot be developed solely on the basis of the type of AF, accounting for the impact of atrial fibrosis in perpetuating AF and its persuasive role in predicting AF ablation failure. However, the result should be interpreted with caution because only two paroxysmal AF studies were included (15, 26).

Low-voltage area ablation in addition to PVI obtained more beneficial clinical outcomes than PVI both in RCT group and non-RCT group, whereas freedom from all-atrial tachycardia recurrence in non-RCT group was higher than RCT group. Part of the reason leading to this discrepancy might be that RCT group enrolled higher proportion of participants with paroxysmal AF (20% vs. 12%). However, the effectiveness of LVA ablation was present predominantly in non-paroxysmal AF patients.

## Limitations

The present study has several limitations. First, any meta-analysis inherits the biases in each of the included studies, and our study is not immune. The characteristics regarding the LVA definition, concept of LVA ablation, endpoint of LAV-guided ablation, radiofrequency power settings, type of 3D mapping system, number of points mapped, ablation targets, and type of high-density mapping catheter were not consistent in all available studies, which creates heterogeneity that has compromised the credibility. Second, both RCT and observational cohort studies were included in this meta-analysis. The application of formal meta-analytic methods to observational studies is controversial. Third, although all of the identified studies adopted Holter monitoring to detect AF/atrial tachycardia recurrence, the duration of Holter monitoring varied widely, ranging from 24 h to 2 weeks. In addition, silent AF/atrial tachycardia recurrence episodes might not have been detected since continuous monitoring was not performed. Fourth, we used unadjusted RR, which does not account for other factor-to-event differences in statistical analysis and might provide an inaccurate impression of the compared effects.

## Conclusion

Low-voltage area-guided substrate modification ablation combined with PVI appears to have a significant beneficial effect of improving freedom from all-atrial tachycardia recurrence, especially in patients with non-paroxysmal AF. Further adequately powered RCT studies are needed to clarify the efficacy and safety of this new substrate modification approach.

## Data availability statement

The original contributions presented in this study are included in the article/Supplementary material, further inquiries can be directed to the corresponding authors.

## Author contributions

All authors have made a significant contribution to the writing, concept, design, execution, or interpretation of the work represented.

## Supplementary material

Supplementary Figure 1 Flow diagram of the review process. LVA, atrial low-voltage areas; PVI, pulmonary vein isolation.

Supplementary Figure 2 Forest plots of all-atrial tachycardia recurrence among patients with LVA-guided substrate modification ablation compared with those who underwent control ablation in six RCT studies. The size of the circle-plotting symbol is proportional to the size of the study. Horizontal lines are the 95% CI. LVA, atrial low-voltage areas; RR, risk ratios; CI, confidence intervals.

Supplementary Figure 3 Sensitivity analysis.

Supplementary Figure 4 Begg’s funnel plot for checking publication bias.
