# Supplementary material for: Efficacy of left atrial low-voltage area-guided catheter ablation of atrial fibrillation: An updated systematic review and meta-analysis
Source: Front Cardiovasc Med. 2022 Nov 17;9:993790. doi: 10.3389/fcvm.2022.993790 (PMC9714681; doi:10.3389/fcvm.2022.993790)
Supplement: Supplementary Figure 1 — Flow diagram of the review process. LVA, atrial low-voltage areas; PVI, pulmonary vein isolation. [file Data_Sheet_1.zip › Supplementary file/Table S1.docx]

Table S1: Procedural characteristics

| Author | Year | Procedure time (min) (Treatment group vs. Control group) | Radiofrequency ablation time (min) (Treatment group vs. Control group) | Endpoint of LAV ablation | Radiofrequency power | 3D mapping system | Mapping points | Mapping catheter | AADs after ablation | The method used to detect arrhythmia recurrence |
| --- | --- | --- | --- | --- | --- | --- | --- | --- | --- | --- |
| Wang X | 2014 | 161 ± 12 vs. 232 ± 10 | 46 ± 9 vs. 134 ± 25 | elimination of all LVZ | 30-35W, 25W for coronary sinus | CARTO3 system | 200-400 | Lasso | AADs were discontinued 3 months after ablation | all-atrial tachycardia>30s, 24h Holter monitoring |
| Rolf | 2014 | 160 (135, 200) vs. 150 (128, 180) | 44 ± 16 vs.39 ± 19 | loss of local capture | 25-40 W, 25 W near the esophagus | EnSite Velocity NavX | 115 ± 35 | Inquiry Optima or Reflexion Spiral or Lasso | AADs were discontinued after ablation, and β-blockers were administered | all-atrial tachycardia>30s, 7-day Holter monitoring |
| Yang G | 2016 | 182 ± 36 vs. 21 1± 34 | - | elimination of all LVZ | 35W, 43°C | EnSite-NavX mapping system | 628 ± 212 | A-Focus catheter; | AADs were discontinued 3 months after ablation | all-atrial tachycardia>30s, 7-day Holter monitoring |
| Jadidi | 2016 | 152 ± 34 vs. 189 ± 51 | 44 ± 19 vs. 28 ± 11 | - | 28-35 W, 20-25W for posterior LA and coronary sinus. | Ensite Velocity-V3 or CARTO3 system | >800 | A-Focus II HD or Lasso-Nav catheter | without AADs treatment | all-atrial tachycardia>30s, 24h Holter monitoring |
| Cutler | 2016 | - | 78 ± 26 vs. 90 ± 34 | - | 25-43W | CARTO3 system | - | - | Amiodarone stopped 2-month and other AADs stopped 3 months postablation | all-atrial tachycardia>30s, 2-week Holter monitoring |
| Yamaguchi | 2017 | 182 ± 26 vs. 158 ± 35 | - | electrogram voltage reduction >50% | 30W, 40°C, 20–25 W near the esophagus | EnSite Verismo version 2.0 | 576 ± 150 | Reflexion HD | AADs were discontinued 6 months after ablation | all-atrial tachycardia>30s, 24h Holter monitoring |
| Yang B | 2017 | 187 ± 53 vs. 211 ± 48 | 60 ± 25 vs. 75 ± 24 | elimination of all LVZ | - | EnSite Verismo version 2.0V | >500 | A-Focus catheter; IBI | AADs were discontinued 3 months after ablation | all-atrial tachycardia>30s, 7-day Holter monitoring |
| Yagishita | 2017 | 267 ± 67 vs. 248 ± 54 | 75 ± 22 vs. 66 ± 19 | elimination of all LVZ | 20-40 W | CARTO3 system | 166 ± 62 | Lasso | AADs were discontinued 3-6 months | all-atrial tachycardia>30s,48h Holter monitoring |
| Mohanty | 2017 | 156 ± 66 vs. 144 ± 78 | 74 ± 34 vs.70 ± 32 | elimination of all LVZ | 40 W | CARTO 3D system | 271 ± 96 | Lasso | AADs were discontinued 12 weeks after ablation | all-atrial tachycardia>30s, 7-day Holter monitoring |
| Kircher | 2018 | 161 ± 43 vs. 164 ± 48 | 37 (26, 55) vs. 41 (29, 51) | loss of local capture | 35 W, 48°C, 25W near the esophagus | Ensite Velocity or CARTO3 system | 120 | Inquiry Optima or Lasso | AADs were discontinued 3 months after ablation | all-atrial tachycardia>30s, 7-day Holter monitoring |
| Kumagai | 2019 | 143 ± 33 vs. 103 ± 39 | 62 ± 23 vs. 41 ± 18 | loss of local capture | 30-40 W, 40°C, 20 W near the esophagus | EnSite NavX system | 524 ± 204 | Optima or Reflexion HD | AADs were discontinued 3 months after ablation | all-atrial tachycardia>30s, 7-day Holter monitoring |
| Nery | 2020 | 305 (262, 360) vs. 273 (240, 342) | 55 (35, 68) vs.50 (43, 63) | loss of local capture | 30-35 W,20-25 W for the posterior wall | CARTO3 system | 1276 ± 691 | Pentaray | AADs were discontinued 6 week after ablation | all-atrial tachycardia>30s, 2-week Holter monitoring |
| Masuda | 2022 | 124 ± 40 vs. 95 ± 33 | - | electrogram voltage reduction >50% | 30 W, 42°C | Rhythmia or CARTO3 system | >800 | Orion or Pentaray | without AADs treatment | all-atrial tachycardia>30s, 24h Holter monitoring |
| Liu | 2021 | - | 85 ±45 vs. 97 ± 43 | loss of local capture | - | CARTO3 system | 452 ± 220 | - | - | all-atrial tachycardia>30s, 24h Holter monitoring |
| Hwang | 2021 | 190 ±34 vs. 187±17 | 71 ± 21 vs. 38 ± 16 | elimination of all LVZ | 30-35W ,20- 25W for posterior wall | CARTO CFAE software module | - | Lasso | without AADs treatment | all-atrial tachycardia>30s, 24h Holter monitoring |
| Suzuki | 2022 | 138± 36 vs. 156 ± 36 | - | elimination of all LVZ or loss of local capture | 25–40 W | EnSite NavX system | ≥500 | Inquiry A focus II | without AADs treatment | AF or atrial flutter>30s, 24h Holter monitoring |

LVA: low atrial low-voltage areas; AADs: antiarrhythmic drugs; AF: atrial fibrillation
