# Supplementary material for: Efficacy of left atrial low-voltage area-guided catheter ablation of atrial fibrillation: An updated systematic review and meta-analysis
Source: Front Cardiovasc Med. 2022 Nov 17;9:993790. doi: 10.3389/fcvm.2022.993790 (PMC9714681; doi:10.3389/fcvm.2022.993790)
Supplement: Supplementary Figure 1 — Flow diagram of the review process. LVA, atrial low-voltage areas; PVI, pulmonary vein isolation. [file Data_Sheet_1.zip › Supplementary file/Table S2.docx]

Table S2: Meta-regression analysis

| Covariates | Coef | t | *P* | 95% CI |
| --- | --- | --- | --- | --- |
| Univariate analysis |  |  |  |  |
| Design | 0.31 | 1.32 | 0.21 | -1.96,0.81 |
| **AF type** | **-0.67** | **-1.80** | **0.10** | **-1.50, 0.15** |
| Rhythm during voltage mapping | -0.12 | -0.44 | 0.67 | -0.72,0.48 |
| The sequence of ablation and mapping | -1.2 | -0.43 | 0.67 | -0.69,0.46 |
| Cutoff of LVA | -0.06 | -0.19 | 0.85 | -0.72,0.61 |
| Follow-up | 0.13 | 0.48 | 0.64 | -0.45,0.71 |
| LAD | 0.21 | 0.76 | 0.45 | -0.38,0.80 |
| Ablation targets | 0.02 | 0.09 | 0.93 | -0.56,0.62 |
| Multivariate analysis |  |  |  |  |
| Design | 0.72 | 1.73 | 0.18 | -0.6,2.04 |
| AF type | -0.52 | -0.76 | 0.50 | -2.67,1.63 |
| Rhythm during voltage mapping | 0.23 | 0.49 | 0.65 | -1.23,1.69 |
| The sequence of ablation and mapping | 0.39 | 0.7 | 0.53 | -1.38,2.17 |
| Cutoff of LVA | -0.42 | -0.91 | 0.43 | -1.87,1.03 |
| Follow-up | 0.04 | 0.08 | 0.94 | -1.48,1.56 |
| LAD | 0.01 | 0.03 | 0.98 | -1.42,1.44 |
| Ablation targets | -0.56 | -0.5 | 0.65 | -4.17,3.04 |

LVA: atrial low-voltage areas, AF: atrial fibrillation, LAD: left atrial diameter
