# Supplementary figures and images for: Efficacy of left atrial low-voltage area-guided catheter ablation of atrial fibrillation: An updated systematic review and meta-analysis
Source: Front Cardiovasc Med. 2022 Nov 17;9:993790. doi: 10.3389/fcvm.2022.993790 (PMC9714681; doi:10.3389/fcvm.2022.993790)

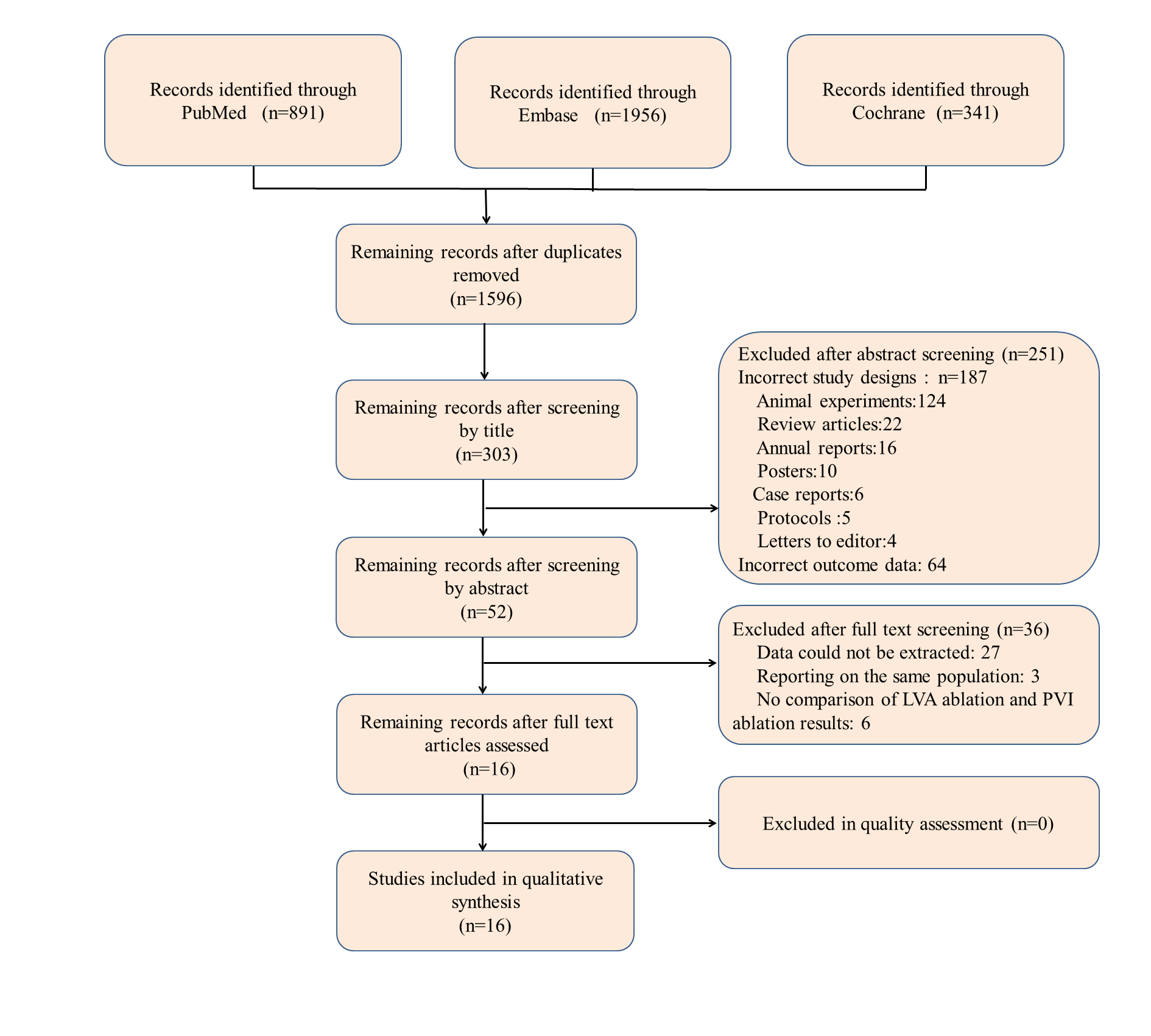

Supplement: Supplementary Figure 1 — Flow diagram of the review process. LVA, atrial low-voltage areas; PVI, pulmonary vein isolation. [file Data_Sheet_1.zip › Supplementary file/Figure S1.tif]

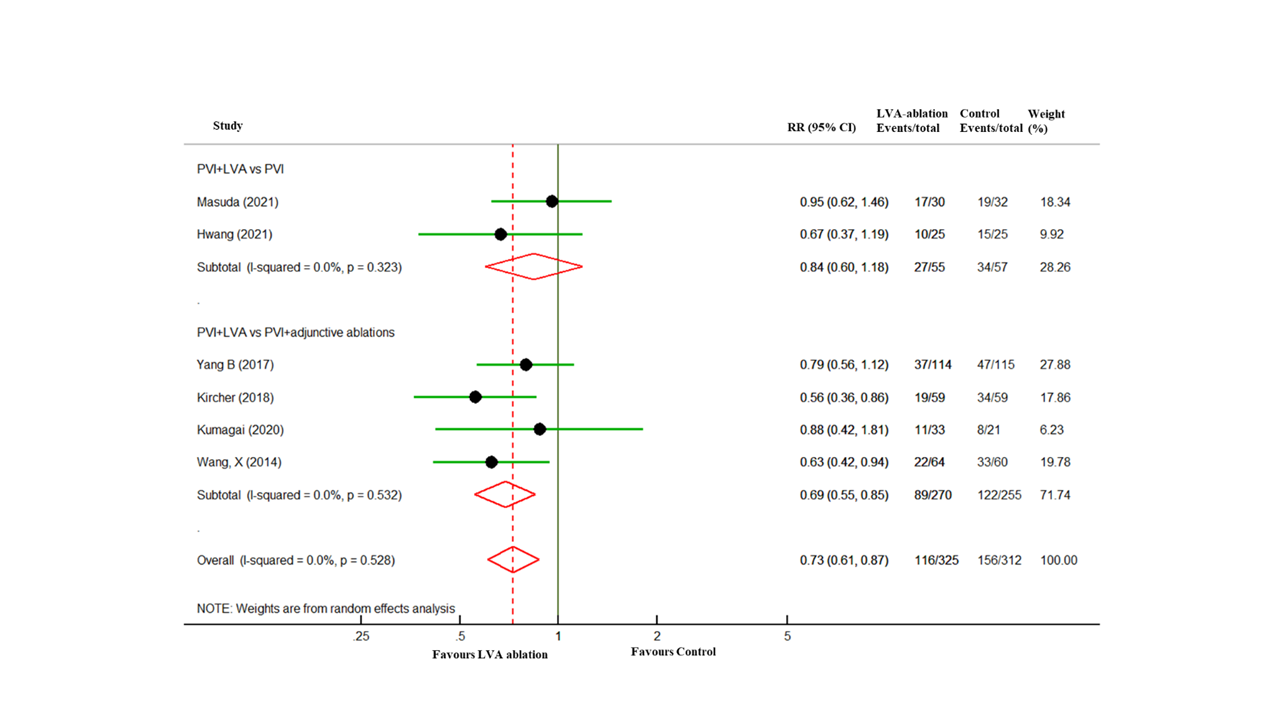

Supplement: Supplementary Figure 1 — Flow diagram of the review process. LVA, atrial low-voltage areas; PVI, pulmonary vein isolation. [file Data_Sheet_1.zip › Supplementary file/Figure S2.tif]

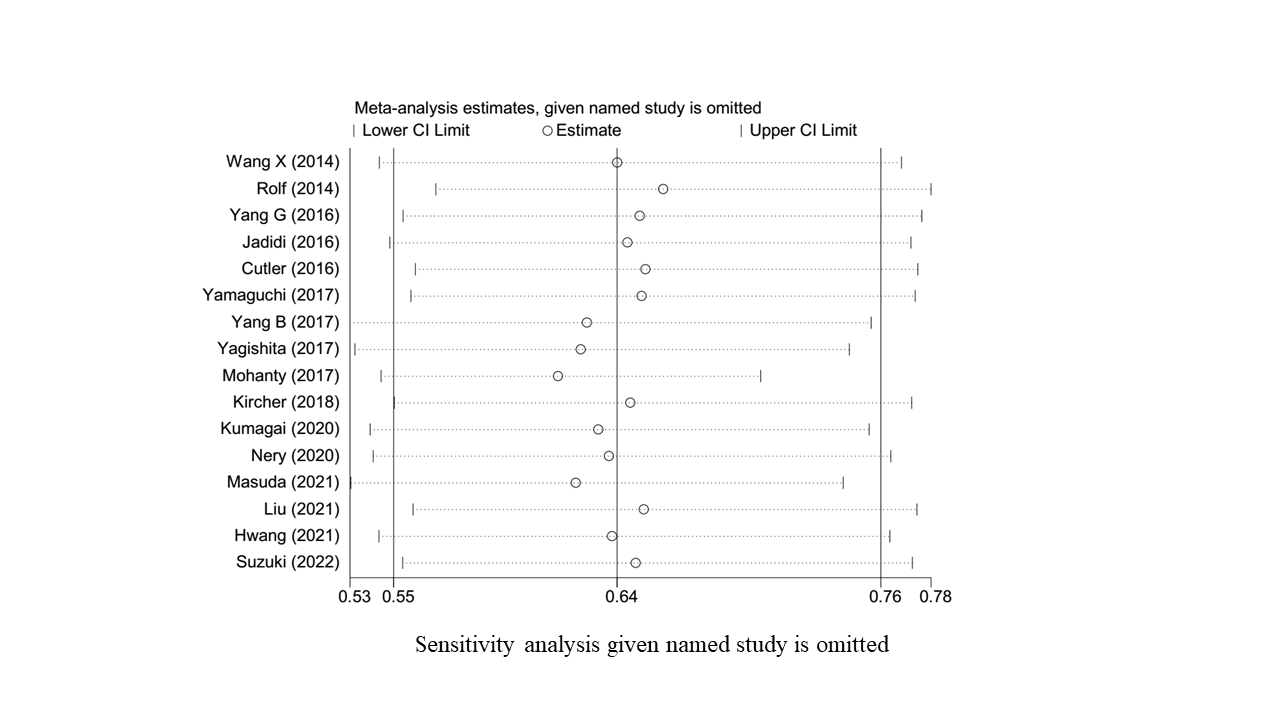

Supplement: Supplementary Figure 1 — Flow diagram of the review process. LVA, atrial low-voltage areas; PVI, pulmonary vein isolation. [file Data_Sheet_1.zip › Supplementary file/Figure S3.tif]

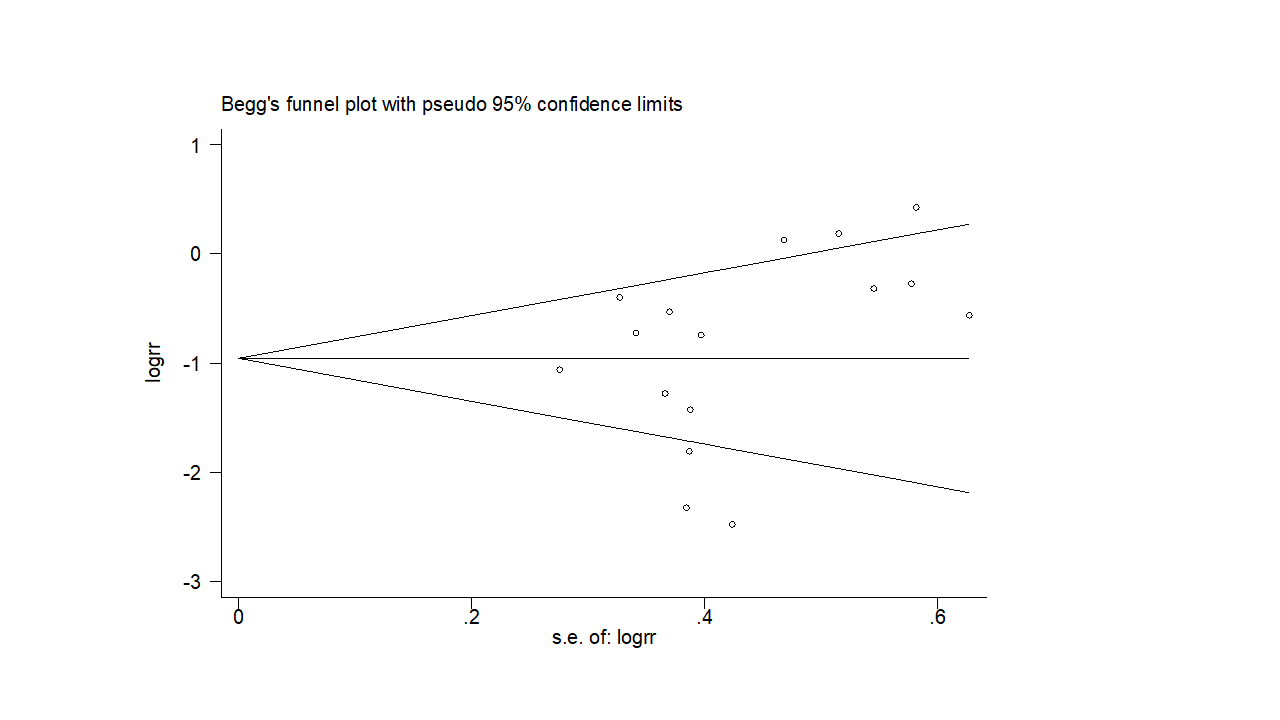

Supplement: Supplementary Figure 1 — Flow diagram of the review process. LVA, atrial low-voltage areas; PVI, pulmonary vein isolation. [file Data_Sheet_1.zip › Supplementary file/Figure S4.tif]
